# Artificial intelligence and medical research databases: ethical review by data access committees

**DOI:** 10.1186/s12910-023-00927-8

**Published:** 2023-07-08

**Authors:** Francis McKay, Bethany J. Williams, Graham Prestwich, Daljeet Bansal, Darren Treanor, Nina Hallowell

**Affiliations:** 1 Population Health Sciences Institute, University of Newcastle, NE2 4AX Newcastle Upon Tyne, UK; 2 National Pathology Imaging Co-operative, Leeds Teaching Hospitals NHS Trust, Leeds, LS9 7TF UK; 3 Yorkshire and Humber Academic Health Science Network, Unit 1, Calder Close, Calder Park, Wakefield, WF4 3BA UK; 4 Department of Pathology, University of Leeds, Leeds, UK; 5 Department of Clinical Pathology, Linköping University, Linköping, Sweden; 6 Center for Medical Image Science and Visualization (CMIV), Linköping University, Linköping, Sweden; 7 The Ethox Centre and the Wellcome Centre for Ethics and Humanities, Nuffield Department of Population Health, University of Oxford, Oxford, OX3 7LF UK

**Keywords:** Artificial intelligence, Medical research databases, Health data repositories, Data access committees, Research ethics committees, Ethical review, Ethical expertise, Public involvement

## Abstract

**Background:**

It has been argued that ethics review committees—e.g., Research Ethics Committees, Institutional Review Boards, etc.— have weaknesses in reviewing big data and artificial intelligence research. For instance, they may, due to the novelty of the area, lack the relevant expertise for judging collective risks and benefits of such research, or they may exempt it from review in instances involving de-identified data.

**Main body:**

Focusing on the example of medical research databases we highlight here ethical issues around de-identified data sharing which motivate the need for review where oversight by ethics committees is weak. Though some argue for ethics committee reform to overcome these weaknesses, it is unclear whether or when that will happen. Hence, we argue that ethical review can be done by data access committees, since they have *de facto* purview of big data and artificial intelligence projects, relevant technical expertise and governance knowledge, and already take on some functions of ethical review. That said, like ethics committees, they may have functional weaknesses in their review capabilities. To strengthen that function, data access committees must think clearly about the kinds of ethical expertise, both professional and lay, that they draw upon to support their work.

**Conclusion:**

Data access committees can undertake ethical review of medical research databases provided they enhance that review function through professional and lay ethical expertise.

## Background

Medical research databases—collections of health information stored for the purpose of research—are an important mechanism by which artificial intelligence (AI) is trained on healthcare data [1–3].^1^ Databases may contain identifiable patient information and, depending on the type in question, could hold a variety of medical information, for instance, genomic data, electronic care records, medical images, etc. To protect individual data subjects’ confidentiality, however, data controllers generally de-identify data prior to sharing it for research. Insofar as de-identifying data is thought to reduce risks to patient privacy, it also limits the need, in many jurisdictions, for ethical review prior to distribution [8–11].

Sharing de-identified medical data for AI research can still raise ethical concerns, however. Studies have shown [12–15], for instance, that multiple ethical issues can arise as a result of the downstream applications of AI. Consequently, there is need for ethical oversight of research using data from medical research databases, even when data is de-identified.^2^

Seemingly, ethics review committees—e.g., Research Ethics Committees (RECS) in the UK, Institutional Review Boards (IRBs) in the USA, etc.—are well placed to undertake this ethical oversight. As Ferretti et al. have argued, however, ethics committees may be “functionally weak” in reviewing big data and AI research due to commonly lacking expertise for assessing the collective risks and benefits of such research [10]. In addition, they may have “purview weaknesses” as exemptions to ethical oversight are often available for projects using de-identified data. Several authors have therefore argued for ethics committee reform, so that they might develop purview and functional strengths for reviewing such research [10, 11, 16]. Pending any reforms, we ask: how else might ethical oversight be provided? We argue here that such work can be done by data access committees (DACs). DACs, we argue, are an appropriate mechanism for ethical review of research applications to medical research databases as they have *de facto* purview of big data and AI projects alongside relevant technical expertise and governance knowledge. As will be shown, they also often take on functions of ethical review. However, like ethics committees, they may have functional weaknesses in relation to ethical review. To strengthen that function, we suggest data access committees must think clearly about their membership structures, and the kinds of ethical expertise they solicit to guide the review process. Most notably, DACs should be mindful to include independent ethical experts, both professional (e.g., bioethicists, data ethicists, etc.) and lay (in the form of patient and public involvement [PPI]).

To make our case we examine the ethical challenges of sharing de-identified medical data via research databases. In doing so, we distinguish between two types of challenges: “upstream” ethical issues impacting individual data subjects’ interests (and which are well protected by existing governance mechanisms), and “downstream” issues impacting collective interests (and which are less well protected). Following that, we make the case for DACs as a strategic mechanism for ethical review regarding the latter, highlighting the importance of independent ethical expertise in the process.

## Main text

### Ethical challenges of sharing de-identified data

Multiple jurisdictions (e.g., the United Kingdom, the United States, Australia, the Netherlands) permit de-identified data sharing from medical research databases with limited or exempted ethical review [9, 13, 17]. As Scott et al. note, such review can include multiple approaches, such as “informing the committee of the project (e.g. by submitting an exemption form) but not submitting an application for review”; “some form of partial or expedited review”; or “bypassing review by an ethics committee completely before commencing the research project” [9]. The reasons for this are well grounded from a research ethics perspective. When data is de-identified, it is understood to reduce risks to data subjects’ privacy [8]. Insofar as a core purpose of research ethics oversight is to protect such interests [18, 19], the need for review is often seen to be obviated.

Many have questioned the degree to which de-identification protects data subjects in an era of big data due to the potential for re-identification [3, 8, 20]. Even where data is robustly de-identified, however, researchers intending to use such data may still encounter ethical issues in their research. For instance, secondary uses of data often entail ethical issues around collective rather than individual interests [6, 16, 18]. Hence, it has been argued that de-identified medical data sharing should be governed by a public health framework, in which the focus is on maximising public benefit and minimising collective harms, as opposed to a research ethics framework in which the focus is on individual risk protection through consent forms, confidentiality agreements, personal information sheets, etc., and which are the specialty of RECs [18].

The downstream risks of de-identified medical data sharing depend on a variety of factors, such as the kind of data (including linked data) being shared, who it is shared with, the purposes for which it is shared, and the specific community interests affected by the research outcomes. That said, general issues have been raised in recent years, particularly around the possibilities of commercialisation and bias.

In relation to the sharing of pathology image data, for instance, such research could exacerbate health inequalities through the privatisation of diagnostic knowledge and technologies [21]. This is a possibility for medical AI research in general insofar as researchers are commonly expected to be commercial organisations, including large technology companies, since they are the ones who possess the advanced engineering expertise and technical resources for bringing medical AI research to market and to clinic in a usable form. As Mittelstadt points out, however, there can be tensions between medical and commercial AI research due to the lack of “common aims and fiduciary duties” [21]. Whereas medicine has long developed professional duties and codes of conduct to facilitate the goal of improving health, the same is not necessarily true of AI, especially in the private sector, where profit incentives may provide a conflict of interest with public health benefits and where AI may, as Spector-Bagdady shows, not be held to the same standard of regulation [6].

The potential for monopolies and unequal distribution of healthcare as a result of proprietary knowledge and tools is not the only avenue for downstream health inequalities, for an additional way is through bias. Often AI bias is understood as datasets that are unrepresentative in terms of relevant demographic and clinical criteria (e.g., health status, age, ethnicity, gender, geographical location, etc.). Multiple authors have noted the impact bias may have for community interests [22, 23]. As Hao notes, however, “bias can creep in long before the data is collected as well as at many other stages of the deep-learning process” [24]. For instance, it can be found in how researchers frame the questions, problems, or categories of research, in how they determine cohort selections in a database, in how they decide what linked data is relevant, and in how they adjust an algorithm’s weights to improve its predictive accuracy. For instance, Obermeyer et al. [25] found that a US healthcare algorithm, which used historical healthcare costs as a proxy for healthcare needs, wrongly classified African Americans as being at lower risk due to having historically lower costs when compared to white Americans. The system affected millions of people and perpetuated racial prejudice by labelling African Americans as requiring less medical care. Though the data here was to some degree biased, so too was the framing of the research and was only improved when the proxy measure was removed.

### Ethical oversight through DACs

To maximize benefits of data sharing while protecting against downstream harms, ethical review of research applications to medical research databases is needed. By “ethical review” we mean oversight through ethical reflection by persons with some form of “ethical expertise” but no conflict of interest in the research.

Ordinarily, ethics review committees provide that oversight. As mentioned, however, de-identified data sharing from medical research databases may be exempt from ethical review due to the limited risks to individual interests that anonymity brings.^3^ Even when ethics committees undertake review of research projects, however, they may still have what Ferretti et al. call “functional weaknesses” in reviewing big data and AI research [10]. Ethics committees are strong when undertaking *ex ante* review of traditional biomedical research (e.g., clinical trials, longitudinal cohort studies, etc.). Here research subjects are directly involved in data collection, demanding ethical protections in the form of consent, confidentiality, etc. Research ethics committees have a strong history of protecting human subjects’ interests in this way. They are less well equipped, however, for overseeing potential downstream harms, largely due to a perceived lack of relevant expertise for judging collective benefits and risks for big data and AI based research [10, 16].

The above weaknesses suggest an “ethics oversight gap” for big data and medical AI research. Responding to that gap, several authors have argued for the need for ethics committee reform [10]. Since it is not clear whether and when such reforms may happen, however, we argue that data access committees (DACs) could provide an alternative site for that ethical review. Unlike ethics committees, DACs generally have technical expertise and governance knowledge around data sharing, making them well-suited for navigating the growing complexities of big data and AI research. Moreover, as data access managers, requests for de-identified medical data come through them by default. Though not all DACs operate the same way, empirical research by Shabani et al. also suggests that many DACs already take ownership of a range of ethical duties, including providing oversight of downstream ethical issues by restricting or flagging culturally or politically controversial uses of data (such as those going counter to prevailing social norms) [27, 28]. In summary, then, DACs are an appropriate site for review, insofar as they have *de facto* purview, functional strengths in the governance of big data and AI research generally, and because they often already take on informal responsibilities for ethical review.

Since not all DACs operate the same way, however, there is need for general advocacy for medical DACs to take on responsibilities for ethical review where that is not already the case. Insofar as they do that, it is also important to note the need for strengthening the review process. This is because, like RECs, DACs face limitations in their capacity for ethical review. As Cheah and Piasecki put it, DACs are in danger “of underestimating or misidentifying potential harms to data subjects and their communities” as they “do not necessarily know what these group harms will be” [29]. Given potential functional weaknesses in terms of judging downstream risks and benefits, DACs should be mindful that they seek relevant ethical expertise to guide their reflections.

The question of what constitutes ethical expertise is a long standing one [30–32]. Though research has not explicitly addressed that question vis-à-vis medical AI research, it has made the case for such expertise in medical research generally. Inspired by that work, we recognise that ethical expertise is possible and desirable for AI research, and that such expertise can take multiple forms, including independent professional ethicists (e.g., bioethicists, legal scholars, critical data scholars, social scientists, etc.) and lay stakeholders (e.g., PPI).^4^ Murtagh et al. [34] have discussed the relevance such interdisciplinary expertise has for responsible data sharing, arguing that, since big data and AI projects are complex, they require a variety of disciplinary and non-disciplinary experts to fulfil important roles such as providing understandings of laws and regulations (in the case of legal scholars), or highlighting relevant contextualising factors that impact research trajectories (in the case of social scientists), etc.

DACs need to specify for themselves the expertise they require for making judgments about data access requests. *Prima facie*, however, the inclusion of lay participants may appear more challenging than professional contributors. The reason comes from the status of professional versus lay expertise in relation to medical AI research generally. Although there is a robust body of professional research in critical algorithm studies and medical AI ethics [14, 35] legitimizing the notion that an emergent disciplinary expertise exists on the ethics of medical AI, there is a relative lack of public awareness and understanding of AI and of its relevance to health research [36]. Related to that, there is also what one might call a “participation deficit” for lay contributors in medical AI research generally, partly due to the novelty of its application, which mirrors a more general participatory gap regarding the use of AI in society [37].

Though we recognize that public understanding and engagement around medical AI may be constrained due to the novelty of its application in society, this does not mean, however, that lay participation on DACs should be overlooked. The benefits of lay representatives in health research are well known [38], suggesting a *prima facie* duty for their inclusion on DACs. Indeed, Health Data Research UK have argued that this should be standard practice [39]. That said, the lack of clarity around what constitutes lay AI ethics expertise or the relevance of lay members to nuanced decision making around data sharing for AI research means further justification is needed. Hence, we highlight here important procedural and substantive justifications in relation to medical AI research.

### Public involvement on DACs

PPI can have value for evidencing procedural fairness insofar as it includes healthcare stakeholders in the decision-making processes around health. This procedural value is important for the ethical oversight of research databases, for if one of the goals of DACs is to maximise the utility of data for public benefit, the question of what constitutes public benefit is one that, procedurally, requires broad public deliberation to determine. Multiple mechanisms exist for deliberating about collective values for AI, from online crowd sourcing to citizen forums [40–42]. Public involvement is a long-standing complementary mechanism to those processes and can continue to be useful within the specific local contexts of research applications. Lay representation on DACs is one way that deliberation can occur for the sake of medical data sharing.

Procedural fairness in decision-making may also have implications for the trustworthiness of database research. As Kerasidou notes, trustworthiness means an individual or organisation evidencing that their decisions are in the best interests of another [43]. Trustworthiness thus shifts the burden of public confidence away from the public toward organisations, which must prove they are worthy of trust. Trustworthiness in regard to medical AI research can be shown by developing algorithms that are lawful, ethical, and robust [44]. Public participation in the reviewing process can further engender that trustworthiness by ensuring that representatives with shared public interests have voice in the decision-making process (which is important where commercial involvement gives reason to question the priorities of the researchers involved). It thereby provides confidence that downstream collective interests have been taken into consideration, which relates to PPI’s substantive contributions.

PPI has substantive value for medical AI research insofar as it can explicate potential collective interests at stake in research applications. Such contributions are relative, however, to the different knowledge and experience PPI members bring. At its broadest level, this means patients and publics. According to Fredriksson and Tritter, patients and publics, though often conflated, bring distinct contributions to PPI discussions: patients offering “sectional” insights based on their experiences as health service users, publics providing a broader societal perspective based on their civic understanding [45].

When examined more closely, however, it becomes apparent that PPI members represent a diverse range of subject positions and collective interests. These may include the general interests of patients as a whole, the specific interests of particular patient communities (such as cancer patients), the interests of community groups defined by demographic characteristics (such as ethnicity, age, or gender), and the broader interests of citizens [40]. The reason they can do this is because they have cultural knowledge about collective interests, which provides them with acquired vigilance allowing them to anticipate relevant community harms and benefits. This vigilance regarding community risks and benefits means they are well placed to anticipate how novel forms of research may impact them. Regarding applications to medical research databases, such “ethical expertise” regarding anticipatory harms and benefits can be used to reflect on possible community impacts, clarify community needs and preferences, and thus guide researchers in how to avoid them.

### Further questions

There are challenges, however, with DACs taking on the functions of ethics review and of the role of PPI in that process. It may be argued, for instance, that not all DACs possess sufficient capacity for such review. Some smaller research groups, for instance, comprise of only one or two people, such as a PI or a post-doctoral researcher, who manage the data access requests [46, 47]. Such groups may lack the ability to outsource ethical review and there could be a conflict of interest if members undertook the review themselves. Such groups may benefit from alternative approaches, therefore. It has been suggested [48], for instance, that central DAC infrastructures could be developed via funding agencies to provide that support. Alternatively, ethical review for smaller DACs might be shifted on to the data applicant, who would provide evidence of having gone through independent ethical review prior to application. The example of smaller research groups is a special case, requiring further deliberation. That issue notwithstanding, appropriately resourced DACs, i.e., those associated with research consortia or institutes, can nonetheless provide a profitable means for filling in the gap left by ethics committees.

It may also be argued that there are alternatives ways in which to address the ethics oversight gap, which could obviate the need for DACs in this regard. Bernstein et al. [26] provide a promising example of an alternative approach in what they call an “Ethics and Society Review board,” which is an *ad hoc* panel devised at Stanford University for the purpose of reviewing the downstream impacts of research. Additionally, ethics awareness training might be provided to raise AI developers’ ethical competencies so that it can inform their work. Fortunately, building ethical reflection into the medical AI research pipeline is not an either/or situation. Moreover, given the complexity of medical AI research, as well as the multiple contexts in which it occurs (involving universities, hospitals, and commercial organisations to different degrees), it is desirable to have multiple means available. DACs, however, are complementary to that and are well placed for providing formal oversight (of the kind usually reserved for RECs) insofar as they are already well-established, have purview over large amounts of medical AI research globally, have strengths in governance and data sharing, and already take on some functions of ethical review. They therefore could be more easily adapted and capitalised on, when contrasted with *ad hoc* panels, which would take time to implement at scale.

Regarding bringing lay representation onto DACs, that could also raise similar kinds of challenges that have been discussed about PPI in other areas. For instance, there is the impracticality of representing all community interests. There is a variety of stakeholders to AI, but it would be unrealistic to survey all interest groups for every data access request. DACs would have to determine membership structures for themselves, though we recognise that there will be times when broad patient and public involvement will suffice to provide oversight on collective interests, and there will be times when more specific community input will be needed. It would be the responsibility of DACs to be alert to the kind of help that is needed and when.

Another issue concerns how to mediate conflict of interests. It is possible PPI members will understand benefits and risks differently. Here we suggest that diversity of viewpoints does not preclude publics from reaching compromise. Insofar as PPI representatives inhabit multiple subject positions, they are able to move beyond sectional interests and recognise the need for trade-offs in the service of a wider public good.

Perhaps most importantly is the advisory nature of public involvement in general, which often entails the possibility for “involvement tokenism,” that is, using PPI as a check box exercise. What “good” public involvement looks like is an ongoing research question [49, 50]. To guard against the ever-present possibility of tokenism, however, we suggest DACs provide opportunities for devolved decision-making to PPI members, for instance, by ensuring all members, including lay members, carry equal weight when deciding whether applications proceed, are rejected, or are sent back for revision.

## Conclusion

Medical research databases are an important means by which AI is trained on health data.Given that researchers may face ethical issues in the application of their work, pre-emptive ethical oversight of research applications is important. Ethics review committees, however, may lack purview or functional strengths when it comes to reviewing big data and AI based medical research. In lieu of ethics committee reforms, DACs are a viable alternative, and are in some ways better placed than ethics committees due to *de facto* purview strengths, technical and governance expertise, and general duties for scientific and ethical review. That said, like RECs, DACs may still exhibit potential functional weakness in their capacity for ethical expertise. Hence, it is recommended that they solicit input in the form of professional and lay ethical experts to strengthen that function. The inclusion of lay participants may appear more challenging than professional contributors, however, due to a lack of public awareness and understanding of AI and a general “participation deficit” for lay contributors in medical AI research. Nonetheless, lay members should continue to be an important cornerstone of ethical reflection due their procedural and substantive contributions.

## Data Availability

No new data were created during this study.

## Abbreviations

AI: Artificial intelligence
DACs: Data access committees
IRBs: Institutional Review Boards
PPI: Patient and Public Involvement
RECs: Research Ethics Committees

## Footnotes

1 “Research database” is the term favoured by the UK’s Health Research Authority [4]. However, they may go under a variety of other names, such as health data repositories, registries, databanks, datalakes, etc. [3, 5–7].

2 We focus here on ex ante or anticipatory ethical review, though much of what can be said would also apply to ex post review.

3 As Bernstein et al. point out  [26], such exclusions may apply whether data is de-identified or not, as in the case of IRBs in the USA, where such assessments are considered outside the purview of the board.

4 Downstream impacts of research may be impacted as much by research quality as research ethics. For instance, a study by Wong et al. [33] shows how a poorly validated proprietary sepsis prediction model implemented in the US predicted onset of sepsis in a way that was “substantially worse” than was reported by its developer. Hence, ethical reflection may also be supported by a range of disciplinary expertise in AI and medicine, and not just professional ethicists or lay contributors.

## References

[1] Health Data Research Innovation Gateway. 2020. https://www.healthdatagateway.org/. Accessed 9 Dec 2021.

[2] Cimino JJ, Ayres EJ (2010). The clinical research data repository of the US National Institutes of Health. Stud Health Technol Inform.

[3] Caplan A, Batra P (2019). The ethics of using de-identified medical data for research without informed consent. Voices Bioethics.

[4] National Research Ethics Service. Ethical review of research databases. 2010. https://www.hra.nhs.uk/documents/376/ethical-review-of-research-databases.pdf. Accessed 13 Jul 2021.

[5] Lee Y-J. Welch Medical Library Guides: Finding Datasets for Secondary Analysis: Research Data Repositories & Databases. 2021. https://browse.welch.jhmi.edu/datasets/nih-data-repositories. Accessed 18 Jan 2022.

[6] Spector-Bagdady K (2021). Governing secondary research use of health data and specimens: the inequitable distribution of regulatory burden between federally funded and industry research. J Law Biosci.

[7] PathLAKE. PathLAKE Research Database - Privacy Notice. 2020. https://www.pathlake.org/wp-content/uploads/2020/09/PathLAKE-Research-Database-privacy-notice-v1.0-090920.pdf. Accessed 5 Aug 2021.

[8] Rothstein MA (2010). Is deidentification sufficient to protect health privacy in research?. Am J Bioeth.

[9] Scott AM, Kolstoe S, Ploem MCC, Hammatt Z, Glasziou P (2020). Exempting low-risk health and medical research from ethics reviews: comparing Australia, the United Kingdom, the United States and the Netherlands. Health Res Policy Sys..

[10] Ferretti A, Ienca M, Sheehan M, Blasimme A, Dove ES, Farsides B (2021). Ethics review of big data research: what should stay and what should be reformed?. BMC Med Ethics.

[11] Friesen P, Douglas-Jones R, Marks M, Pierce R, Fletcher K, Mishra A (2021). Governing AI-Driven Health Research: Are IRBs Up to the Task?. Ethics Hum Res.

[12] Fullerton SM, Lee SS-J (2011). Secondary uses and the governance of de-identified data: lessons from the human genome diversity panel. BMC Med Ethics..

[13] Ienca M, Ferretti A, Hurst S, Puhan M, Lovis C, Vayena E (2018). Considerations for ethics review of big data health research: a scoping review. PLoS ONE.

[14] Morley J, Machado CCV, Burr C, Cowls J, Joshi I, Taddeo M (2020). The ethics of AI in health care: a mapping review. Soc Sci Med.

[15] Vayena E, Blasimme A, Cohen IG (2018). Machine learning in medicine: addressing ethical challenges. PLoS Med.

[16] Samuel G, Chubb J, Derrick G (2021). Boundaries between research ethics and ethical research use in artificial intelligence health research. J Empir Res Hum Res Ethics.

[17] El Emam K, Rodgers S, Malin B (2015). Anonymising and sharing individual patient data. BMJ.

[18] Ballantyne A (2019). Adjusting the focus: a public health ethics approach to data research. Bioethics.

[19] Hemminki E (2016). Research ethics committees in the regulation of clinical research: comparison of Finland to England, Canada, and the United States. Health Res Policy Sys.

[20] Sweeney L. Simple Demographics Often Identify People Uniquely. 2000. https://dataprivacylab.org/projects/identifiability/paper1.pdf. Accessed 18 May 2022.

[21] Mazer BL, Paulson N, Sinard JH (2020). Protecting the pathology commons in the digital era. Arch Pathol Lab Med.

[22] Ibrahim H, Liu X, Zariffa N, Morris AD, Denniston AK (2021). Health data poverty: an assailable barrier to equitable digital health care. Lancet Digit Health.

[23] Kaushal A, Altman R, Langlotz C (2020). Geographic distribution of US cohorts used to train deep learning algorithms. JAMA.

[24] Hao K. This is how AI bias really happens—and why it’s so hard to fix. 2019. https://www.technologyreview.com/2019/02/04/137602/this-is-how-ai-bias-really-happensand-why-its-so-hard-to-fix/. Accessed 18 May 2022.

[25] Obermeyer Z, Powers B, Vogeli C, Mullainathan S (2019). Dissecting racial bias in an algorithm used to manage the health of populations. Science.

[26] Bernstein MS, Levi M, Magnus D, Rajala B, Satz D, Waeiss C. ESR: ethics and society review of artificial intelligence research. 2021. 10.1073/pnas.2117261118 PMC8719852 34934006

[27] Shabani M, Dove ES, Murtagh M, Knoppers BM, Borry P (2017). Oversight of genomic data sharing: what roles for ethics and data access committees?. Biopreserv Biobank.

[28] Shabani M, Thorogood A, Borry P (2016). Who should have access to genomic data and how should they be held accountable? Perspectives of Data Access Committee members and experts. Eur J Hum Genet.

[29] Cheah PY, Piasecki J (2020). Data access committees. BMC Med Ethics.

[30] Weinstein BD (1994). The possibility of ethical expertise. Theoret Med.

[31] Rasmussen LM (2005). Ethics expertise: history, contemporary perspectives, and applications.

[32] Yoder SD (1998). The nature of ethical expertise. Hastings Cent Rep.

[33] Wong A, Otles E, Donnelly JP, Krumm A, McCullough J, DeTroyer-Cooley O (2021). External validation of a widely implemented proprietary sepsis prediction model in hospitalized patients. JAMA Intern Med.

[34] Murtagh MJ, Blell MT, Butters OW, Cowley L, Dove ES, Goodman A (2018). Better governance, better access: practising responsible data sharing in the METADAC governance infrastructure. Hum Genomics.

[35] Gillespie T, Seaver N. Critical Algorithm Studies: a Reading List. Social Media Collective. 2016. https://socialmediacollective.org/reading-lists/critical-algorithm-studies/. Accessed 25 Jan 2022.

[36] Castell S, Robinson L, Ashford H (2018). Future data-driven technologies and the implications for use of patient data.

[37] RSA (2018). Artificial intelligence: real public engagement.

[38] Staniszewska S (2009). Patient and public involvement in health services and health research: a brief overview of evidence, policy and activity. J Res Nurs.

[39] Health Data Research UK. Building trust in data access through public involvement in governance: survey findings and recommendations from HDR UK’s Public Advisory Board. UK: Health Data Research; 2021.

[40] McKay F, Williams BJ, Prestwich G, Treanor D, Hallowell N (2022). Public governance of medical artificial intelligence research in the UK: an integrated multi-scale model. Res Involv Engagem.

[41] O’Doherty K, Einsiedel E (2013). Public engagement and emerging technologies.

[42] Rahwan I (2018). Society-in-the-loop: programming the algorithmic social contract. Ethics Inf Technol.

[43] Kerasidou A (2017). Trust me, I’m a researcher!: the role of trust in biomedical research. Med Health Care and Philos.

[44] Hleg AI (2019). Ethics guidelines for trustworthy AI: independent high-level expert group on artificial intelligence.

[45] Fredriksson M, Tritter JQ (2017). Disentangling patient and public involvement in healthcare decisions: why the difference matters. Sociol Health Illn.

[46] Shabani M, Dyke SOM, Joly Y, Borry P (2015). Controlled access under review: improving the governance of genomic data access. PLoS Biol.

[47] Shabani M, Borry P (2016). “You want the right amount of oversight”: interviews with data access committee members and experts on genomic data access. Genet Med.

[48] Shabani M, Knoppers BM, Borry P (2015). From the principles of genomic data sharing to the practices of data access committees. EMBO Mol Med.

[49] Liabo K (2020). Public involvement in health research: what does ‘good’ look like in practice?. Res Involv Engagem.

[50] McCoy MS, Jongsma KR, Friesen P, Dunn M, Neuhaus CP, Rand L (2018). National standards for public involvement in research: missing the forest for the trees. J Med Ethics.

